# An integrated theoretical‐experimental approach to accelerate translational tissue engineering

**DOI:** 10.1002/term.2346

**Published:** 2017-04-05

**Authors:** Rachel H. Coy, Owen R. Evans, James B. Phillips, Rebecca J. Shipley

**Affiliations:** ^1^ CoMPLEX University College London London UK; ^2^ Department of Mathematics Columbia University USA; ^3^ Biomaterials and Tissue Engineering University College London London UK; ^4^ Department of Mechanical Engineering University College London London UK

**Keywords:** peripheral nerve repair, mathematical modelling, translational research

## Abstract

Implantable devices utilizing bioengineered tissue are increasingly showing promise as viable clinical solutions. The design of bioengineered constructs is currently directed according to the results of experiments that are used to test a wide range of different combinations and spatial arrangements of biomaterials, cells and chemical factors. There is an outstanding need to accelerate the design process and reduce financial costs, whilst minimizing the required number of animal‐based experiments. These aims could be achieved through the incorporation of mathematical modelling as a preliminary design tool. Here we focus on tissue‐engineered constructs for peripheral nerve repair, which are designed to aid nerve and blood vessel growth and repair after peripheral nerve injury. We offer insight into the role that mathematical modelling can play within tissue engineering, and motivate the use of modelling as a tool capable of improving and accelerating the design of nerve repair constructs in particular. Specific case studies are presented in order to illustrate the potential of mathematical modelling to direct construct design. Copyright © 2016 The Authors Journal of Tissue Engineering and Regenerative Medicine Published by John Wiley & Sons Ltd.

## 1 Introduction

The rate of progress in the highly promising field of tissue engineering is hindered by the need for scientists to consider a wide array of different factors when finalizing designs for clinical use, ranging from material and cell types to spatial configurations. These parameters have the potential to impact clinical efficacy, and currently are investigated predominantly via experimental techniques. The use of mathematical modelling could speed up and streamline the process of identifying optimal design parameters, provided that the models can be parameterized effectively. The search for a bioengineered solution to the problem of peripheral nerve regeneration, in the form of nerve repair construct (NRC) design, offers a prime opportunity to demonstrate the potential of theoretical techniques in tissue engineering.

**Figure 1:**
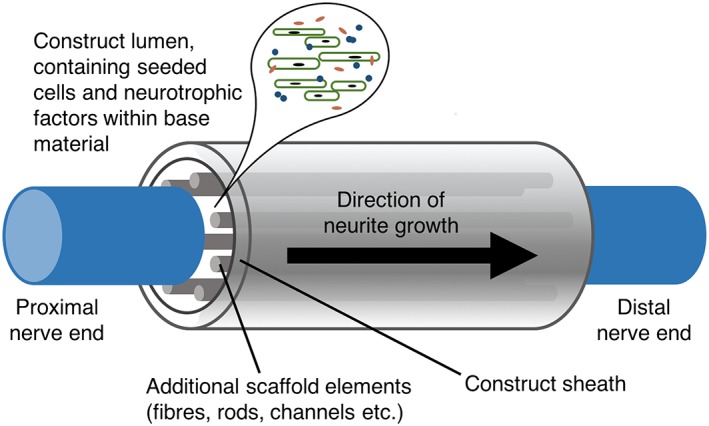
Nerve repair construct (NRC) composition. NRCs consist of a sheath containing materials and therapeutic cells. These inner core components are designed to deliver the chemical and mechanical cues necessary to guide and promote effective nerve regrowth. The outer sheath of an NRC requires mechanical strength and the ability to protect the growing tissue within

The development of NRCs that support neurite regrowth after injury has so far resulted in plenty of innovative research, and a wide range of different construct design specifications have been trialled, both *in vivo* and *in vitro* (Angius *et al*., 2012; Faroni *et al*., 2015; Gu *et al*., 2011; Figure 1). Although significant progress in understanding has been made, there has been little translation of experimental constructs to clinical application, as reviewed in Scheib and Höke (2013) and Guena *et al*. (2012). Further refinement of construct design is needed in order to emulate and exceed the levels of efficacy demonstrated by ‘gold‐standard’ autografts in a clinical setting. This is a challenging task, not only because researchers seeking to improve nerve construct design are faced with a large number of variables to consider, but also because it is important that cells and materials are used in a cost‐effective manner, and that the manufacturing process is feasible on a clinical scale.

Over 70 different materials have been used as part of NRC designs in studies thus far, including both biologically derived materials, such as collagen, and synthetic materials (Angius *et al*., 2012). As well as the core scaffold materials, there are many other factors that are important to consider during the process of designing an NRC, including sources of therapeutic cells, and chemical and topographical cues, which have been described in further detail in previous reports (Angius *et al*., 2012; Bell and Haycock, 2012; Deumens *et al*., 2010). Determining the best way of organizing and assembling all of these components is experimentally challenging.

Mathematical modelling offers a way to standardize, streamline and accelerate the nerve construct development workflow by providing additional rationale for experimental design decisions. Modelling does provide its own challenges, such as the dilemma of initial mathematical model choice, the need for sufficient quality and quantity of data for good parameterization, and the requirement for experimental validation of results. This paper motivates the use of mathematical modelling to inform NRC design, outlines a possible workflow to help overcome these challenges, and provides examples of the application of similar theoretical techniques to other tissue‐engineering problems. Examples of preliminary NRC mathematical model case studies are also presented, demonstrating the type of interactions that can be incorporated into a mathematical framework and how the resulting outputs have the capacity to direct NRC design.

## 2 Motivation for mathematical modelling in NRC design

Nerve repair construct design has so far been predominantly informed by experimental results and ‘scientific intuition’ alone. Experimentalists often make decisions regarding the quantities and spatial arrangement of construct prototype design factors, such as therapeutic cells and scaffold material, based almost entirely upon previously published experiments. However, these existing studies are not standardized, as demonstrated by the diversity in approach of the experiments reviewed in Angius *et al*. (2012) and Nectow *et al*. (2012), which increases the difficulty in deciding which design elements are optimal for a particular repair scenario.

**Figure 2:**
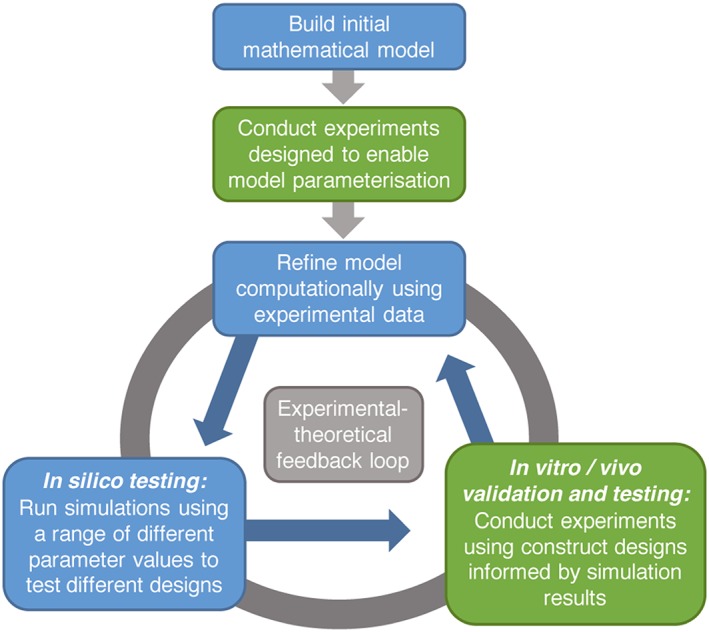
Proposed theoretical‐experimental tissue‐engineering design workflow. An initial mathematical framework, parameterized via either existing or specifically collated experimental data, can be improved iteratively. Model outcomes can inform the design and direction of experiments, the results of which can in turn refine and expand the model, allowing for accelerated progress with a more clearly defined direction

The integration of mathematical modelling into the NRC design workflow has the potential to alleviate these issues and thereby transform the field. It is proposed that mathematical modelling could be incorporated into the current NRC design workflow via the use of simulations completed as an additional step, prior to the design of *in vitro* and *in vivo* experiments (Figure 2). Once a model has been constructed, this preliminary *in silico* investigative step would offer an extremely quick and cheap way of testing the efficacy of possible NRC designs, and generating design hypotheses that can then be tested experimentally, or to help in the preparation of a target profile. Subsequent experiments would be carefully planned and executed to test the most promising designs found computationally, identify design parameters for constructs, and simultaneously act to validate the model, allowing iterative improvement. Although some existing data could be used to help validate mathematical models, this paper advocates the use of experiments specifically designed for this purpose. This will allow choices of outcome assessment methods and experimental set up that correspond directly to the model.

Foremost among the problems intrinsic to the current purely experimental approach is the sheer number of different variables that need to be considered when designing an NRC. The results of mathematical model simulations run using a variety of different input parameters corresponding to different combinations of design variables would indicate which design configurations are most likely to produce the best outcomes and provide much needed guidance for experimentalists faced with a multitude of factors to consider. Investigating the huge number of possible different design specifications via experiments alone would be expensive and time consuming; therefore, utilizing simulations in this manner would not only give direction to future research but also allow experimentalists to make use of their resources in a more efficient and cost‐effective manner.

Furthermore, computational simulations would allow researchers to investigate the relative importance of different variables. By systematically differing the parameters incorporated into a model that reflects these variables, and monitoring the resulting outcomes, quantitative sensitivity analyses can be carried out. This will inform researchers of the margin of error in the NRC manufacturing process; if altering a certain variable, such as the density of seeded cells at a specific point in the construct, by a small amount results in large changes in the simulated efficacy of the construct, then bioengineers need to control that variable more carefully during the manufacturing process. Conclusive evidence could also arise from simulations that a certain parameter has relatively little impact upon the efficacy of an NRC. Thus, research could be effectively prioritized, helping to accelerate advances in the field by reducing the time spent investigating parameters that would have little bearing on the clinical efficacy of the constructs, and consequently allowing research teams to allocate and spend their resources more wisely by investigating those parameters that have the greatest impact.

Importantly, the use of mathematical modelling also conforms to the principles of the 3Rs of humane animal research (Russell and Burch, 1959). The replacement of animal experiments with other scientific methods, and the overall reduction of the number of animals used in experiments, are long‐standing ethical goals for experimental researchers. In the field of NRC design, the number of *in vivo* experiments, and consequently the number of animals needed, could be reduced dramatically via the use of *in silico* simulations. The aforementioned method of streamlining research via simulation‐aided identification of particularly important parameters will also allow researchers to maximize the benefits gained from *in vivo* experimentation. Therefore, the motivations for the introduction of modelling are ethical as well as scientific and financial.

## 3 Mathematical modelling approaches to tissue‐engineering challenges

Tissues and organs grown in laboratories can be used to replace those that have been damaged through trauma, disease or ageing, or alternatively maintained *in vitro* for drug screening programs and toxicity tests. The exact biochemical and biomechanical composition of engineered tissues must be tightly controlled to ensure that they function correctly. This requires detailed knowledge of the interactions that take place between the many different chemical and structural variables involved, which often occur at vastly different speeds and across different temporal and spatial scales. The integration of discrete and continuum spatio‐temporal mathematical models, refined using experimental data, can improve understanding of these processes and therefore aid the design of bioengineered tissue.

Thus far, attempts to use theoretical models to explore bioengineered tissue scenarios have varied widely in both their approach and their level of success. However, some studies have certainly produced informative results that demonstrate the capacity of a theoretical approach to influence the direction of experimental design, through the identification of important design factors. One example is a mathematical framework developed by Sanz‐Herrera *et al*., 2009 which allowed them to simulate the *in vivo* regeneration of bone tissue using a scaffold implant, with the aim of optimizing the performance of such scaffolds through increased understanding of their behaviour (Sanz‐Herrera *et al*., 2009). A multi‐scale approach was used to couple macro‐ and micro‐scale interactions, enabling the simulations to capture both the organ‐level interaction between the scaffold and the surrounding bone, as well as bone regeneration and scaffold decomposition occurring on a smaller scale within the scaffold domain. The study analysed how perturbing different design parameters, such as scaffold stiffness, affected percentage bone regeneration and scaffold resorption indices over time. The simulation results predicted that the rate of bone regeneration within implanted scaffolds increases with greater scaffold stiffness and mean pore size, and with the incorporation of scaffold cell pre‐seeding. These outcomes demonstrate how mathematical modelling can effectively identify highly sensitive parameters, which induce significant changes in outcome when altered, in contrast to parameters that are less likely to have a dramatic effect when modified. Ordering the parameters into a hierarchy of sensitivity and influence using inferences based upon modelling predictions could help bioengineers prioritize and streamline the tissue‐engineering testing and design process.

Mathematical and computational modelling has also been used in the field of *in vitro* tissue engineering with the goal of improving the design of bioreactors, which are used to cultivate and direct tissue growth through the delivery of nutrients to cells, coupled with the introduction of mechanical cues. Lawrence *et al*. used computational fluid dynamics software to explore the impact of bioreactor geometry, cell‐seeded scaffold porosity, and differing inlet and outlet patterns on fluid flow within the bioreactors (Lawrence *et al*., 2009). The goal was to identify factors that could cause suboptimal fluid distributions resulting in non‐uniform shear stress distributions and poor nutrient delivery, which adversely affect the calibre of the regenerated tissue. The quality of the fluid distribution was characterized by the residence time distribution (RTD) function, which provides an approximation of the amount of time molecules in the fluid spend within a bioreactor (Fogler, 2005). Experiments were performed to validate the simulation results, using bioreactors constructed to correspond to the simulation bioreactor geometries. Tracer concentrations, RTD profiles and pressure drops across porous bioreactors were determined experimentally and compared with the theoretical results. In some cases, these experiments confirmed the validity of the model; in others, they suggested that additional factors such as compression of porous structures needed to be incorporated into the equations used. This demonstrates the iterative process of mathematical model development, parameterization and validation against experimental data, which is ongoing in this example.

Analysis of simulation fluid pressure values indicated that the size of pores within the scaffold influences the pressure gradients and consequently the flow regime achieved within the bioreactor. Consequently, porosity is a factor that may need to be carefully optimized by tissue engineers in order to ensure sufficient fluid flow and nutrient delivery. Although further inquiry into the relationship between different flow distributions and important factors such as nutrient consumption would be beneficial in this case, the study demonstrates how relatively simple mathematical functions such as the RTD, alongside the careful analysis of variables such as stress and pressure, can help to rule out poor design choices within bioengineering projects.

The validation of theoretical models is essential to verify their ability to provide accurate results with the capacity to direct bioengineered tissue design. However, this can often prove difficult. Sanz‐Herrera *et al*., 2009 used an actual scaffold microstructure geometry obtained via micro‐computed tomography instead of an idealized geometry, but struggled to execute proper quantitative validation of the model against experimental data (Sanz‐Herrera *et al*., 2009). This was partly because the existing literature could not provide values for all of the input parameters, which needed to be designated in the model prior to running the simulations. This underlines the importance of designing theoretical models with parameters that can be assigned values gleaned either from experiments designed especially for this purpose, or else from existing experimental data. Designing mathematical frameworks in this way helps to identify and potentially limit sources of uncertainty and error, and consequently improves the relevance of any design‐related hypotheses that such frameworks may generate.

Although interdisciplinary scientific research has recently become more common, often projects involving both experimental and computational/mathematical approaches fail to integrate the two methods fully into one symbiotic workflow. Although models certainly have the capacity to suggest potential design improvements, these are rarely taken forward and implemented in the wet lab. True interdisciplinary research would involve a recursive relationship between the two approaches, with simulation results providing insight into the kinds of experiments needed to maximize progress, and highlighting lines of enquiry that may otherwise remain untapped. It will be important in the future to properly integrate mathematical modelling into such an interdisciplinary workflow, although it is likely that this ‘true’ interdisciplinary approach would require a significant overhaul in the structuring and funding of research, enabling greater flexibility to facilitate this kind of cross‐departmental collaboration. However, despite the issues raised in this section, work published thus far has certainly built a convincing case for the utility of interdisciplinary methods in the field of tissue engineering (Byrne *et al*., 2007; Lawrence *et al*., 2009; Sanz‐Herrera *et al*., 2009; Shipley and Waters, 2011; Williams *et al*., 2013).

## 4 Case studies in NRC design

In order to illustrate how mathematical modelling can be incorporated into the process of NRC design in an effective manner, two case studies will now be outlined. Together, these preliminary modelling examples act as a simple proof of concept by establishing some of the methods available for such theoretical endeavours, as well as indicating how these results will be useful in improving the design of NRCs.

**Figure 3:**
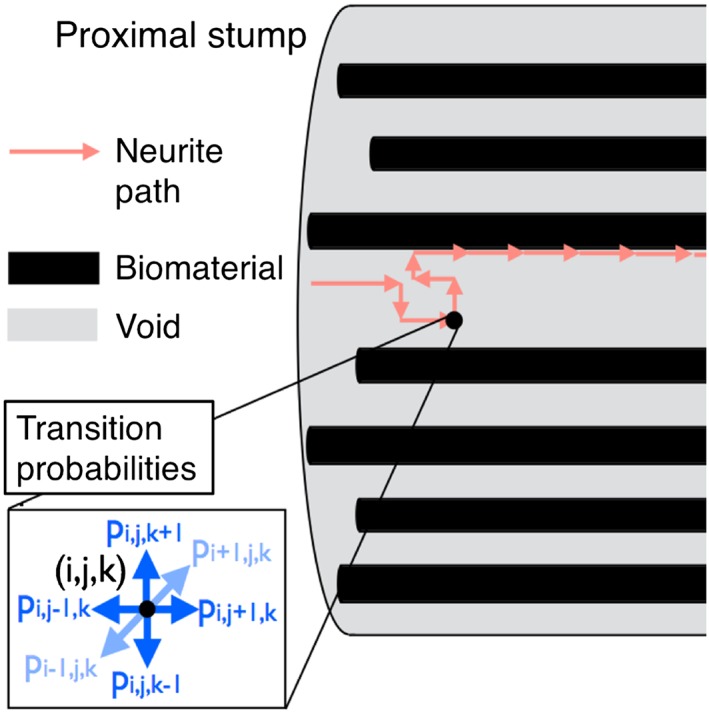
Schematic of the modelling set‐up. Axonal growth is initiated at the proximal stump, and the path of neurite growth is determined by a probabilistic model. Both the temporal and 3D spatial domain are discretized, and at each time step neurites sprout from the sites located at the proximal end of the construct with a prescribed probability. Once a sprout has formed at any one of these sites, it proceeds to grow according to a Markovian random walk, with transition probabilities as depicted in the figure. These transition probabilities may capture directional biases, reflective of neurite response to underlying mechanical and biochemical cues, and can vary in space and time. Further, the transition probabilities mimic neurite interaction with material boundaries between the internal construct fluid space and specified regions of biomaterial placed within the construct, for example through no penetration conditions. The time interval for movement between two neighbouring grid points is set as a constant (1 mm/day), and this gives rise to an average neurite growth rate that varies both spatially and temporally. The transition probabilities reflect the propensity of growing neurites to follow the directional cues provided by the biomaterial content of the NRC, and indeed provide the opportunity to refine the level of physical cue (controlled, for example, through material density) required to support regeneration within a defined time frame

**Figure 4:**
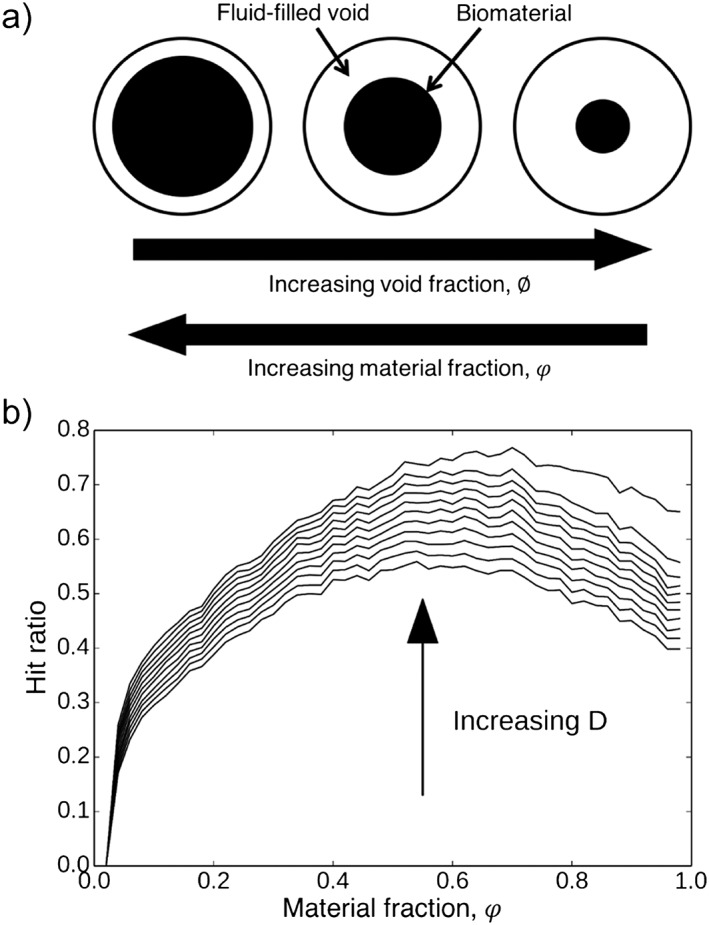
(a) Depiction of void fraction. Note that ϕ  +  φ  =  1. (b) Simulated hit ratios, the proportion of neurites generated at the proximal stump that exceeded a target longitudinal distance down the construct within a 2‐week period, for a range of longitudinal distances *D*, as a function of material fraction φ. The plot illustrates that increasing the material fraction from zero, and consequently decreasing the void fraction, results in more growth‐promoting directional material cues, thus facilitating greater hit ratio values. However, after a certain critical value increases in material fraction result in decreasing hit ratios, as the space available for neurite growth becomes limited, as represented by small void fraction values

The first model describes the regeneration of neurons within a cylindrical NRC (Figure 1), via the use of a 3D random walk process determined by a spatially dependent probability distribution (Figure 3; Evans *et al*., 2014). Parameters within the model, such as the neurite sprouting probability and growth rate, were defined using knowledge of previously conducted experiments (Kim *et al*., 2012). Model simulations can be used to shed light upon the way that alterations in NRC void fraction affect neuronal growth. Void fraction *ϕ* is defined in the context of this model as the volume fraction of space occupied by fluid, matrix and cells within the construct, and φ is defined as the fraction of material, which is usually implemented in the form of sheets or rods, so that ϕ+φ=1. A void fraction of 1 corresponds to a tube containing no additional biomaterial components, with *ϕ* decreasing as the material content increases. Note that the model still allows for neurite growth, unaided by the presence of such internal material structures, in this case. The degree of neurite growth was tracked using a hit ratio function, defined as the proportion of neurites generated at the proximal stump that exceeded a target longitudinal distance down the construct within a 2‐week period. A higher hit ratio would indicate more neurite growth and consequently a potentially improved functional outcome. Figure 4 depicts the simulated hit ratio values for different longitudinal distances *D* from the proximal end of the construct, as the material fraction φ varies. The current literature does not provide sufficient longitudinal, time‐dependent growth data to verify these simulations. Experimentalists could, however, verify the simulation results and test model‐generated hypotheses, focusing on comparisons between designs with void fraction values highlighted as particularly viable within the model and a *ϕ* = 1 , *φ* = 0 control, and thus potentially improve the efficacy of their constructs without an excessive number of trial‐and‐error style experiments. The parabolic nature of these curves suggests that there is an optimal value for the void fraction that maximizes the hit ratio at each point along the construct, which arises from the need to balance the delivery of sufficient material cues via implanted biomaterial for good cell growth, with providing enough space for regeneration.

The model can also be used to investigate the effects that different spatial arrangements of material sheets have upon neuronal growth. For example, Georgiou *et al*. previously conducted *in vivo* experiments using NRCs manufactured using two different configurations of their aligned cellular collagen‐based material EngNT, to compare the distributions of neuronal regeneration achieved after 4 weeks (Georgiou *et al*., 2013). One arrangement consisted of two rolled sheets, or rods, of EngNT packed within the construct sheath, whereas the other design also incorporated two sheets but placed them loosely to form concentric layers lining the construct. The final axon densities measured at different spatial locations within the rod device were marginally greater than those found in the concentric sheet device. Although informative, this study looks at only a tiny number of the many different possible arrangements of EngNT available; investigating even a modest proportion of these potential designs experimentally would be expensive and time consuming, motivating the alternative use of a neuronal growth mathematical model.

**Figure 5:**
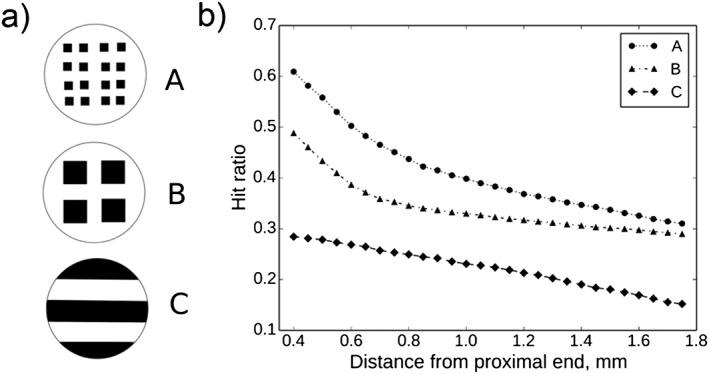
(a) Cross‐sectional arrangements of EngNT rods, A–C. (b) Simulated hit ratios for different longitudinal distances *D*, with ϕ  =  0.5, for the three different EngNT arrangements. From these simulation results, it appears that a higher accessible surface area of material within a construct would aid regeneration. Any other possible cross‐section could also be investigated using this model framework

Simulations were run to explore the sensitivity of the hit ratio to alterations in the cross‐sectional arrangement of EngNT rods whilst maintaining a constant total void fraction value (Figure 5), which was set in this case as *ϕ* = 0.5, approximately in the region of the optimum value identified in Figure 4. Design A, which partitioned the material into the smallest units of those tested, thus increasing surface area of material available to guide regeneration, resulted in a consistently greater simulated hit ratio along the length of the construct, suggesting that future experiments could begin by investigating arrangements similar to design A.

This mathematical framework has not been fully validated against experimental data and could certainly be improved, for example by coupling the discrete stochastic model described here to a secondary continuum model, in order to capture the interplay between the growth of the neurites and the spatio‐temporal distribution of trophic factors. However, it is sufficient to demonstrate the advantages of being able to quickly simulate and compare the neurite distributions achieved by a huge number of different designs. The use of such a model to identify the most likely design candidates for successful growth, and those with little chance of achieving clinical outcomes close to the autologous graft ‘gold standard’, would give a much needed sense of direction to subsequent experimental studies.

The second case study demonstrates how the quest to improve initial nerve ingrowth, from the proximal stump of an injury site into the NRC, provides another opportunity for the generation of practical, testable hypotheses via mathematical modelling. Georgiou *et al*. found that the number of sprouting axons that grow into the proximal end of an EngNT‐based NRC was approximately half that of an autograft, suggesting that design features of the EngNT construct limit the final degree of successful neuronal regeneration (Georgiou *et al*., 2013). After the initial short‐distance sprouting phase, EngNT was shown to provide sufficient support for good consistent growth along the length of the construct, with about 70% of the neurites that managed to enter the proximal end of the construct also successfully reaching the distal end of the construct (Georgiou *et al*., 2013). The incorporation of phosphate glass fibres has shown potential for promoting short‐distance, initial regeneration from the proximal stump (Joo *et al*., 2012). These fibres can provide mechanical support to EngNT within constructs, as well as haptotactic cues for the initial axonal sprouts.

Simulation results from the neuronal regrowth model above suggest that there is an optimal porosity for neurite regeneration, which can be achieved in this setting by adjusting the number and size of glass fibre rods used. A relationship between the porosity *ϕ* (equivalent to void fraction), the number of glass fibre rods used within a construct *N*, their diameter *d*_*r*_ and the diameter of the NRC *d*_*c*_ has been proposed:

(1) $$\;N=\frac{d_{c}^{2}}{d_{r}^{2}}\left(1-\phi \right)$$

**Figure 6:**
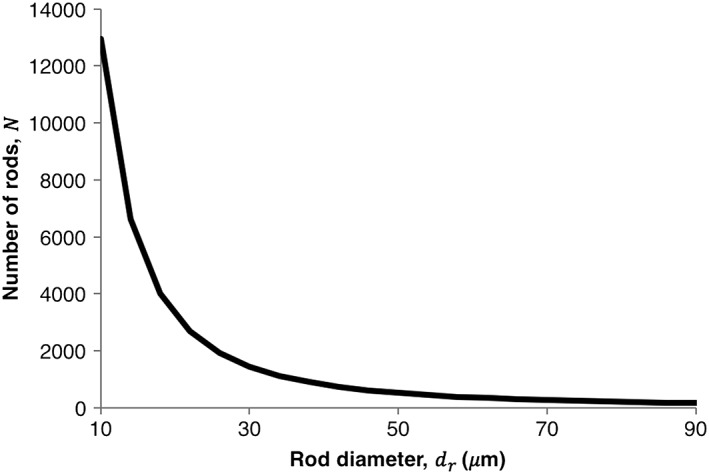
Possible (*N*, *d*_*r*_) pairs to achieve a porosity of 0.6 for a construct of diameter 1.8 mm. These values were calculated using Eqn (1). Although any of these combinations could be used to create a construct with the proposed optimal porosity of 0.6, a final (*N*, *d*_*r*_) pair could be selected from this plot based upon practical considerations, such as the feasibility of manufacture

Such an equation allows experimentalists to calculate the number or size of the rods to use within their constructs in order to achieve the previously calculated optimal porosity value. For example, to achieve porosity *ϕ* = 0.6 in an NRC with radius *d*_*c*_ = 0.9 mm, experimentalists could use a range of (*N*, *d*_*r*_) pairs, as exhibited by Figure 6. Recent experiments by Kim *et al*. used 750 phosphate glass fibres of mean diameter 14.99 μm arranged in a 0.8‐mm‐diameter construct, obtaining a porosity of 0.73 (Kim *et al*., 2012). Further experimentation could test whether a porosity of 0.6, attained using associated (*N*, *d*_*r*_) pairs (Figure 6), does in fact offer improved neurite growth. This is another example of how feasible mathematical relationships such as this, which can be validated experimentally, can indicate which NRC designs are most likely to achieve increased clinical efficacy.

## 5 Conclusion

The examples outlined in the previous section demonstrate the potential for mathematical modelling to accelerate progress in the design of bioengineered nerve repair solutions. However, far more complex, accurate and informative models could be built in conjunction with experimental data. For example, the amount of vascularization that occurs along the length of NRCs is likely to affect their level of efficacy. Therefore, incorporating the process of angiogenesis into a mathematical model, parameterized using experimental data, could potentially be of use. Other aspects such as cell viability will also need to be considered and taken into account. Luckily, unlike experiment‐based research, a mathematical modelling approach allows for the integration of many different interactions and processes, and the control of a large number of parameters, which can be assigned according to a smaller number of carefully designed and focused experiments.

It is expected that the exact designs that result in the best clinical efficacy will differ to those that achieve good results in preclinical experiments, which are often conducted *in vivo* using rats or mice. The design changes needed to take into account the variation in biological and mechanical cues between the clinical and preclinical scenarios could also be informed by a mathematical modelling approach. These differences could be incorporated into a modelling framework by adjusting the corresponding model parameters, as informed by experimental and clinical evidence.

Eventually, modelling frameworks could even be used to develop stratified or patient‐specific NRCs. The medical profile of a patient, including factors such as age and the presence of diseases such as diabetes, will also feasibly alter the values of parameters used within an NRC model, such as the neurite growth rate. Feeding these patient‐specific details into a mathematical model will allow the design of personalized NRCs, with a greater chance of clinical success.

The use of mathematical and computational modelling would both accelerate and provide direction to the process of NRC design. The potential for large reductions in the number of animal‐based experiments, and the consequential financial savings, also make up compelling arguments for the integration of modelling into the design workflow. Experiments would be required to parameterize and validate any mathematical framework, but probably fewer than for a purely experimental approach, and thus the eventual coupling of theoretical and experimental techniques has the capacity to considerably improve the efficiency of NRC design. Similar integrated interdisciplinary methods could be implemented to great effect in other areas of tissue engineering.

### Funding

This work was supported by EPSRC and a doctoral training grant SP/08/004 from the British Heart Foundation (BHF) to Rachel Coy. Rachel Coy is also supported through the UCL CoMPLEX doctoral training programme.

## Conflict of interest

The authors declare no conflicts of interest.
